# Engineering and stable production of recombinant IgE for cancer immunotherapy and AllergoOncology

**DOI:** 10.1016/j.jaci.2017.12.986

**Published:** 2018-04

**Authors:** Silvia Crescioli, Giulia Chiaruttini, Silvia Mele, Kristina M. Ilieva, Giulia Pellizzari, Daniel I.R. Spencer, Richard A. Gardner, Katie E. Lacy, James F. Spicer, Andrew N.J. Tutt, Gerd K. Wagner, Sophia N. Karagiannis

**Affiliations:** a St John's Institute of Dermatology, School of Basic & Medical Biosciences, King's College London, London, United Kingdom; b NIHR Biomedical Research Centre at Guy's and St Thomas's Hospitals and King's College London, London, United Kingdom; c Breast Cancer Now Unit, School of Cancer & Pharmaceutical Sciences, King's College London, Guy's Cancer Centre, London, United Kingdom; d Ludger, Culham Science Centre, Abingdon, United Kingdom; e School of Cancer & Pharmaceutical Sciences, King's College London, Guy's Hospital, London, United Kingdom; f Breast Cancer Now Toby Robins Research Centre, Institute of Cancer Research, London, United Kingdom; g Department of Chemistry, Faculty of Natural & Mathematical Sciences, King's College London, London, United Kingdom

To the Editor:

AllergoOncology, the emerging discipline of cancer immunology aiming to exploit features of allergy-related immunity to treat tumors,^1^, ^2^, ^3^ has catalyzed the development of tumor-specific IgE mAbs as powerful alternatives to commonly used therapeutic IgGs.^4^, ^5^, ^6^ IgE, which is associated typically with the pathogenesis of allergic responses and is known for Fc-mediated protective effects in parasitic infection clearance, presents exciting opportunities to unleash previously untapped immune mechanisms and effective antitumor surveillance when focused against cancer antigens. The antitumor efficacy of IgE has been demonstrated in numerous studies,^1^, ^2^, ^3^ and an early clinical trial of the first-in-class antitumor IgE in oncology is open (NCT02546921, www.clinicaltrials.gov). A major impediment in the field relates to lack of efficient cloning and production strategies for recombinant IgE at high enough yields for preclinical and clinical studies.

We aimed to develop stable expression application to generate recombinant IgE, as exemplified by an antibody recognizing the melanoma-associated antigen chondroitin sulfate proteoglycan 4 (CSPG4). Our strategy incorporates seamless cloning, selection, and fast antibody production at high yields (Fig 1, *A*). To prevent promoter silencing, we developed a novel dual-plasmid system containing Ubiquitous Chromatin Opening Element (UCOE) sequences located upstream of the transgene promoter.^7^ We isolated the coding sequences of anti-CSPG4 IgE heavy and light chains from a previously described (pVITRO1-CSPG4 IgE/k) vector (Fig 1, *B*)^4^ and cloned these into 2 UCOE vectors (UCOE-CSPG4-HC[ε] and UCOE-CSPG4-LC[κ]; Fig 1, *C*) by using Polymerase Incomplete Primer Extension (PIPE) cloning. UCOE enables higher transfection efficiency and higher proportions of medium- and high-expressing transfectomas than pVITRO1 (see Fig E1 in this article's Online Repository at www.jacionline.org). Vectors were linearized before transfection to allow correct integration into the host genome, and transgene-expressing cells were selected. The choice of Expi293F cells as hosts was based on human-like glycosylation profiles, ability to grow in suspension, high-density and serum-free conditions, and characteristics crucial for expediting production, scaling up, and adaptability to good manufacturing practice conditions. We adapted Expi293F cells from suspension to adherent growth conditions and *vice versa*. Adherent cells were transfected and seeded in selection medium to promote host genome integration of exogenous DNA. Resistant cells were cloned by limiting dilution. We designed a cell-based flow cytometric method to detect functional IgE recognizing natively expressed antigens to screen antibody-secreting clones (see Fig E1). Clones with high antibody expression were amplified and readapted to grow in high-density suspension cultures for antibody harvesting.

**Fig 1:**
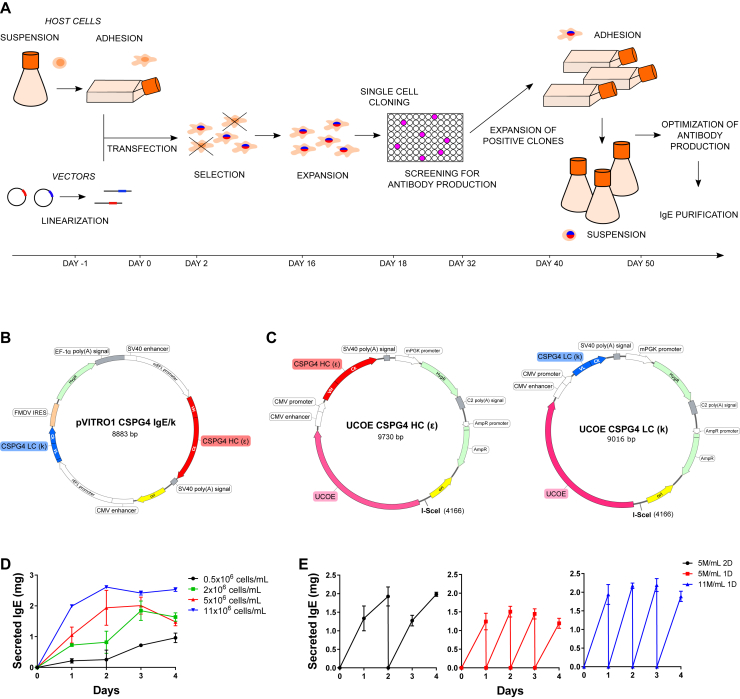
Development of a stable platform for the expression of recombinant IgE. **A,** Flow chart summarizing the development of stable cell lines expressing anti-CSPG4 IgE. **B** and **C,** pVITRO1-CSPG4-IgE/κ vector (Fig 1, *B*) and UCOE-CSPG4-HC(ε) and UCOE-CSPG4-LC(κ) vector (Fig 1, *C*) maps. To optimize antibody production, Expi-CSPG4-IgE cells were cultured in different conditions, and IgE secretion and cell viability were monitored daily. **D,** Secreted IgE in cultures seeded at 0.5, 2, 5, or 11 × 10^6^ cells/mL in fresh medium. **E,** Secreted IgE in cultures seeded at 5 × 10^6^ or 11 × 10^6^ cells/mL in 25 mL of fresh medium and reseeded at the initial concentration every day (5M/mL 1D, 11M/mL 1D) or every 2 days (5M/mL 2D). Data in Fig 1, *D* and *E*, represent means ± SEMs of 4 independent experiments.

After selecting the highest-expressing clone, we optimized culture conditions to maximize IgE production and minimize time and resources. We observed a slow decrease in specific daily antibody productivity consistent with cell growth rate and consumption of culture medium nutrients. This productivity decrease was due to nutrient depletion in the medium rather than cell density (see Fig E2 in this article's Online Repository at www.jacionline.org).

To maximize yields, we tested different seeding Expi-CSPG4 IgE cell concentrations in fresh medium, measuring secreted antibody daily for 5 days. As expected, higher starting cell concentrations yielded faster and greater antibody production, with cells seeded at 11 × 10^6^ cells/mL generating 2 mg/d (Fig 1, *D*).

Using high cell concentrations (5 × 10^6^ and 11 × 10^6^ cells/mL), which place cells under stress, we analyzed production consistency over time by passaging every 2 days at 5 × 10^6^ cells/mL (5M/mL 2D) or every day at 5 × 10^6^ cells/mL (5M/mL 1D) or 11 × 10^6^ cells/mL (11M/mL 1D), replacing medium at every passage. After 4 days, all conditions yielded consistent antibody production (Fig 1, *E*). The 11M/mL 1D and 5M/mL 2D conditions yielded similar production per passage. However, 11M/mL 1D resulted in the highest production per day (see Fig E3 in this article's Online Repository at www.jacionline.org), suggesting this is optimal for reducing resources and time.

IgE production reached yields of up to 87 mg/L/d (83 ± 4 mg/L/d [mean ± SD]), with the ability to repeat the process with the same cells at least 3 times without losing production efficiency. Yields in 4 days in small shaking flask cultures were approximately 33-fold greater than the most optimal 14-day stable IgE production recorded in shaking flask conditions and 13-fold higher than 14-day IgE production reported by using bioreactors.^4^ Optimized high-density conditions allowed maximized yields and substantially reduced medium volumes, achieving 2 mg/25 mL/d, with similar yields scaling down to 15-mL and up to 300-mL cultures.

Different culture conditions can affect antibody quality and structural and functional properties, including posttranslational modifications, such as glycosylation.^8^ This is highly pertinent for IgE based on larger size and higher glycosylation levels compared with IgG. We performed structural, glycosylation, and functional analyses comparing affinity chromatography–purified antibodies from high- and low-density cultures with IgE produced with the previous pVITRO method.^4^

Size-exclusion HPLC showed very similar antibody main peak profiles (Fig 2, *A*). Lectin blot and liquid chromatography–mass spectrometry (LC-MS) glycosylation analyses revealed no significant differences among IgE produced with our method (Fig 2, *B* and *C*), particularly with regard to oligomannose structures, removal of which is reported to abrogate anaphylaxis.^9^ LC-MS glycosylation analyses showed a reduction in MAN5 oligomannose structure in new preparations compared with pVITRO IgE. Antibodies from all conditions showed binding characteristics comparable with those of target antigen on A375 melanoma cells and rat basophilic leukemia RBL-SX38 cells expressing human FcεRI (Fig 2, *D*). All preparations triggered significant and comparable levels of mast cell degranulation when cross-linked by polyclonal anti-IgE or target antigen on CSPG4^high^ melanoma cells (Fig 2, *E*). The hapten-specific NIP-IgE cross-linked by polyclonal anti-IgE, but not by CSPG4^high^ cells, triggered significant degranulation. These suggest that different preparations and density conditions preserve receptor recognition and antibody potency. Importantly, reduced MAN5 in the new IgE is insufficient to affect IgE binding or functionality.

**Fig 2:**
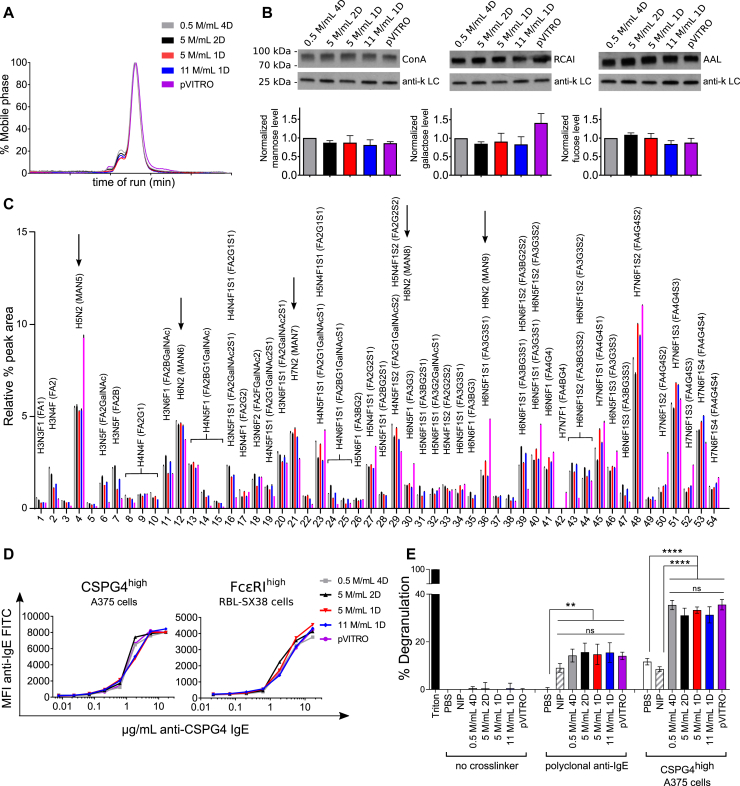
Structural and functional characterization of IgE produced under different conditions. IgE was purified from supernatants of Freestyle293F-CSPG4 IgE (pVITRO) or Expi-CSPG4 IgE cultured at 0.5 × 10^6^ for 4 days, (0.5M/mL 4D), 5 × 10^6^ for 2 days (5M/mL 2D), 5 × 10^6^ for 1 day (5M/mL 1D) or 11 × 10^6^ cells/mL for 1 day (11M/mL 1D). **A,** Structural characterization was performed by using HPLC. **B,** Lectin blotting was performed by using Con-A, AAL, and RCAI. Images show 1 representative experiment. Graphs show densitometric analyses normalized by means of kappa light chain Western blotting. Data represent means ± SEMs of 2 independent experiments. **C,** LC-MS glycosylation analysis. The graph represents relative percentage areas for each ultra-HPLC chromatogram peak of procainamide-labeled N-glycan released from each sample. Data represent means ± SDs. Monosaccharide compositions assigned to peaks: *F*, fucose; *H*, hexose; *N*, N-acetylhexosamine; *S*, sialic acid. Possible N-glycan structures shown in brackets: *A*, antenna; *F*, fucose; *G*, galactose; *MAN*, mannose; *S*, sialic acid. **D,** IgE-binding kinetics to CSPG4 antigen (A375 cells) and FcεRI (RBL-SX38 cells). **E,** IgE-mediated degranulation of RBL-SX38 mast cells measured in negative control (no cross-linker), positive control (polyclonal anti-IgE), and using a CSPG4-expressing tumor cell line to trigger cross-linking of anti-CSPG4 IgE-FcεRI complexes. Data represent means ± SEMs of 4 independent. **P < .01 and ****P < .0001. *ns*, Not significant.

Therefore we present a novel process for serum-free production of IgE with comparable structural and functional characteristics to the previous pVITRO method but at higher yield and in less time than any documented stable platforms and with less resources than transient systems (see Methods and Table E1 in this article's Online Repository at www.jacionline.org). This offers new opportunities to expedite the design of novel therapeutic antibodies with enhanced effector functions suitable for basic and translational research, scaling up, process development, and manufacturing for clinical trials of IgE therapy in patients with cancer. Rapid generation of IgE antibodies recognizing allergens, cancer antigens, or parasitic targets can find direct applications for exploring multifaceted functions of IgE in allergy, oncology, and AllergoOncology.

## Figures and Tables

**Fig E1:**
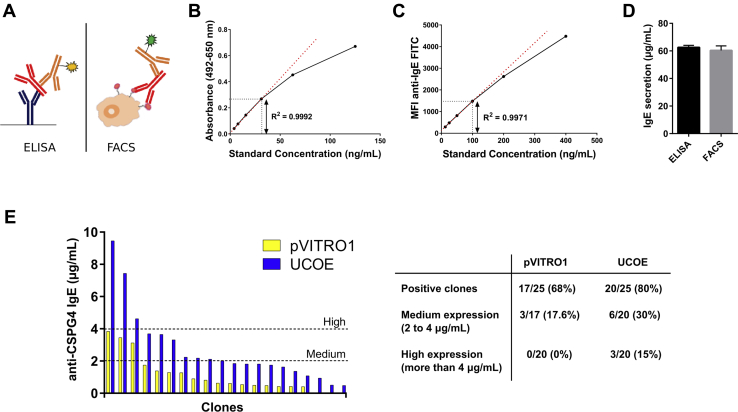
Detection of functionally active antibodies in culture supernatants by using flow cytometry. **A,** Schematic depicting the principle for antibody detection in cell-culture supernatants by using a classical sandwich ELISA (ELISA) and our novel cytofluorometry-based assay (flow cytometry). **B** and **C,** Standard curves showing ranges of linearity *(dotted lines)* for ELISA (Fig E1, *B*) and for flow cytometry (Fig E1, *C*). **D,** Comparison between IgE concentrations calculated with ELISA and flow cytometry. *Error bars* represent SEMs of 3 independent experiments. **E,** Screening of clones transfected with pVITRO1-vector or UCOE-vector system graph representing secreted anti-CSPG4 IgE detected with flow cytometry. Clones secreting between 2 and 4 μg/mL anti-CSPG4 IgE were considered medium-expressing clones, and those that produced greater than 4 μg/mL were considered high-expressing clones. The *right panel* summarizes absolute numbers and percentages of different antibody expression levels.

**Fig E2:**
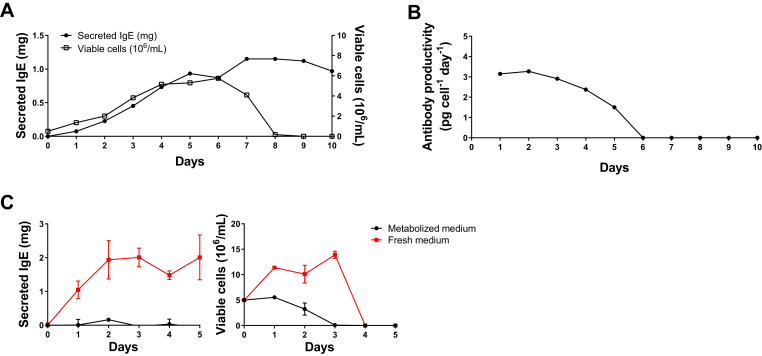
Culture medium conditions for optimal antibody production. To optimize antibody production, Expi-CSPG4-IgE cells were cultured in different conditions, and IgE secretion and cell viability were monitored daily. **A,** Secreted IgE and viable cells in cultures seeded at 0.5 × 10^6^ cells/mL in fresh medium. **B,** Antibody-specific productivity calculated from Fig E2, *A*. Graphs in Fig E2, *A* and *B*, represent one of 2 independent experiments. **C,** Secreted IgE *(left panel)* and viable cells *(right panel)* in cultures seeded at 5 × 10^6^ cells/mL in fresh or metabolized medium. Secreted IgE is normalized on secreted IgE at day 0. Data represent means ± SEMs of 4 independent experiments.

**Fig E3:**
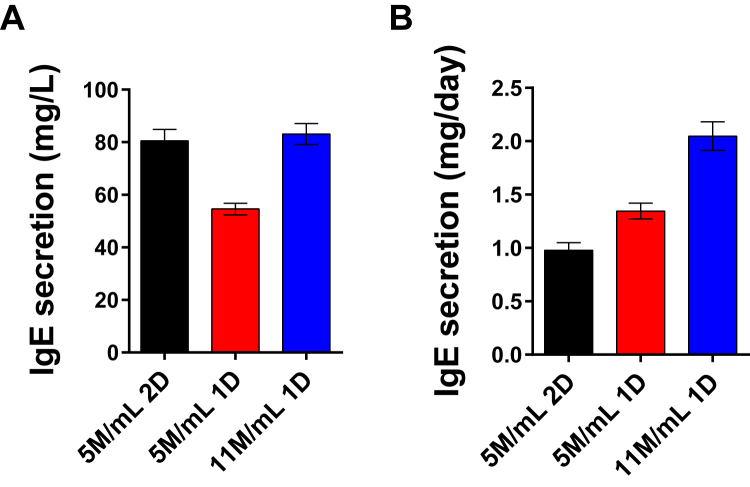
Analysis of data reported in Fig 1, *E*. The bar chart represents the average yield per passage (in milligrams per liter; **A**) or average yield per day (in milligrams per day; **B**). Data represent means ± SEMs of 4 independent experiments.

**Table E1:** Main features of the proposed method

Expression host	Expi293F (human origin, suitable for high-density and serum-free culture conditions)
Vector	Dual system with monocistronic UCOE-vectors (strong CMV promoter for high expression, UCOE sequence able to prevent promoter silencing)
Expression system	Stable
Vessel and physical conditions	Shaking flasks (125 rpm), 37°C, 8% CO_2_
Medium	Expi293 Expression Medium, serum free
Yield	Up to 87 mg/L
Volume	Suitable for small and upscaled cultures (from 15-300 mL)
Cell density	Up to 11 × 10^6^ cells/mL
Time to set up the stable platform	Approximately 50 days
Time for antibody expression	Twenty-four hours (2 mg in 25 mL of culture)
Cost for setup of the stable platform	PIPE cloning, adhesion-based and small-volume stable transfection with Lipofectamine 2000 and FACS-based screening of clones, make the development of the stable platform cost-effective compared with the production of antibody by using transient transfection
Cost for antibody expression	High yield in a small volume of Expi293 Expression Medium (up to 87 mg of IgE for 1 L of medium)

**Table E2:** PIPE cloning primers

Primer name	5′-3′ sequence
Isolation of ε heavy chain	
H F	AGTTAACGGCCGGCCATGGACTGGACCTGGAGGATCCTC
E R	TGCAGCTCGAGTCATTTACCGGGATTTACAGACACCGCT
Isolation of κ light chain	
K F	AGTTAACGGCCGGCCATGTTGCCATCACAACTCATTGGG
K R	TGCAGGTCGACCTAACACTCTCCCCTGTTGAAGCTC
UCOE vector linearization in 3 segments (U1, U2, and U3)	
U1 F	GTCGACCTGCAGGCATGCAAG
U1 R	CGTTGTCAGAAGTAAGTTGG
U2 F	CCAACTTACTTCTGACAACG
U2 R	AGCACTAACGAAGTAGAGGG
U3 F	CCCTCTACTTCGTTAGTGCT
U3 R	GGCCGGCCGTTAACTTAACTAAC

**Table E3:** PIPE cloning PCR conditions

Temperature (°C)	Time (s)				
	ε heavy chain	κ light chain	U1	U2	U3
98	10	10	10	10	10
98	1	1	1	1	1
60	5	5	5	5	5
72	23	9	43	42	34

## Methods

### Cell lines

Expi293F cells were cultured in Expi293 Expression Medium in suspension and shaking conditions (125 rpm) at 37°C in an 8% CO_2_ atmosphere, according to the manufacturer's instructions, seeding them at 0.5 × 10^6^ cells/mL every 4 days. A375 melanoma cells were cultured in adhesion in Dulbecco modified Eagle medium (DMEM) high glucose supplemented with GlutaMAX (Gibco, Grand Island, NY) and 10% FBS. RBL-SX38 rat basophilic leukemia cells (engineered to express the human FcεRI)^E1^ were cultured in adhesion.

### CSPG4 IgE expression vectors

The pVITRO1-CSPG4-IgE/k vector (Fig 1, *B*) was previously developed in our group.^E2^ The dual UCOE-vector system (UCOE-CSPG4-HC[ε] and UCOE-CSPG4-LC[κ]; Fig 1, *C*) was developed by using PIPE cloning, as described previously.^E2^ Briefly, the coding sequences of the CSPG4 IgE heavy and light chains were isolated from pVITRO1-CSPG4-IgE/k vector, and UCOE Mu-H vector (Merck Millipore, Burlington, Mass) was linearized in 3 portions by using the primers shown in Table E2 and the PCR conditions reported in Table E3. The PCR products were then digested for 2 hours at 37°C with *Dpn*I (New England Biolabs, Ipswich, Mass) to eliminate template DNA, and 2 separate mixes were prepared by using the 3 vector portions and 1 heavy or light chain. The 2 mixes were incubated overnight at room temperature and then used to transform chemically competent One Shot TOP10 bacteria (Invitrogen, Carlsbad, Calif), according to the manufacturer's instructions.

### Development of stable IgE-expressing Expi293F cells

The Expi293F cell line (Gibco) was cultured, according to the manufacturer's instructions, in Expi293 Expression Medium (Gibco). One day before transfection, 2 × 10^5^ cells were seeded in a 24-well plate in DMEM, high glucose supplemented with GlutaMAX (Gibco) and 10% FBS to simultaneously adapt them to grow in adherence conditions and achieve 90% confluence at the point of transfection. UCOE-CSPG4-HC(ε) and UCOE-CSPG4-LC(k) were linearized by using I-SceI (New England Biolabs), according to the manufacturer's instruction. Adherent cells were transfected with either pVITRO1-CSPG4-IgE/k or the 2 linearized UCOE vectors with 0.8 μg of DNA, 2 μL of Lipofectamine 2000 (Invitrogen), and 600 μL of Opti-MEM I Reduced Serum Medium (Gibco), according to the manufacturer's instructions. A mock transfection (without vector) was performed as a control.

Sixteen hours after transfection, the cells were split 1:10 and seeded in complete medium (DMEM, high glucose supplemented with GlutaMAX [Gibco] and 10% FBS). The following day, medium was replaced with selection medium (complete medium supplemented with 200 μg/mL Hygromycin B; Sigma, St Louis, Mo). Cells were then cultured, replacing the selective medium every 3 days until the point when all the cells from the mock transfection died. The selection-resistant cells were then amplified and cloned by limiting dilution in selection media. After 2 weeks, the clones were screened for secretion of anti-CSPG4 IgE in the supernatant, and positive clones were amplified and then adapted back to growth in suspension and shaking conditions in Expi293 Expression Medium. Different clones were frozen, and the best clone (Expi-CSPG4 IgE) was used for optimization of antibody production.

### CSPG4 IgE production with stable Freestyle293-CSPG4 IgE cells

CSPG4 IgE was produced, culturing Freestyle293-CSPG4 IgE according to our previously described method (pVITRO1).^E2^

### ELISA assay for detection and quantification of IgE in cell supernatants

Ninety-six-well plates were coated overnight at 4°C with 10 μg/mL polyclonal rabbit anti-IgE (Dako, Glostrup, Denmark) in 0.2 mol/L carbonate-bicarbonate buffer (pH 9.4) to quantify anti-CSPG4 IgE in cell-culture supernatants. Plates were incubated in blocking buffer (PBS [0.01 mol/L phosphate buffer, 0.0027 mol/L KCl, and 0.14 mol/L NaCl], pH 7.4, supplemented with 1% BSA) for 1 hour at room temperature, washed 3 times with washing buffer (PBS plus 0.05% Tween-20), and incubated with supernatants or with the IgE antibody standard at different dilutions for 2 hours at room temperature. All conditions were tested in duplicates. Plates were washed 3 times and incubated with 10 μg/mL goat anti-human Fcε-HRP (Sigma-Aldrich) in blocking buffer for 1.5 hours at room temperature. After 3 washes, samples were treated with 50 μL of 0.5 mg/mL o-phenylenediamine dihydrochloride substrate (Sigma-Aldrich) in peroxide substrate buffer (Pierce, Rockford, Ill) for 5 minutes at room temperature. After addition of 50 μL per well of Stop Solution (1 mol/L HCl), plates were read at 492 nm (reference wavelength, 540 nm) on a FLUOstar Omega Microplate Reader (BMG Labtech, Ortenberg, Germany).

### Flow cytometry–based assay for detection and quantification of antigen-specific IgE in cell supernatants

This method is based on detection in cell supernatants of IgE antibodies that specifically bind to CSPG4^high^ tumor (A375 melanoma) cells. A375 cells were detached by using PBS supplemented with 5 mmol/L EDTA and resuspended in PBS supplemented with 2% FBS (fluorescence-activated cell sorting [FACS] buffer). For this assay, we used 10^5^ cells per sample in a final volume of 100 μL. For quantification of antigen-specific IgE, a standard curve was prepared with 1:2 serial dilutions of CSPG4 IgE. For analysis of CSPG4 IgE in cell-culture supernatants, 0.1 μL of supernatant was prepared in FACS buffer, for a final dilution of 1:1000. Primary antibodies were incubated for 30 minutes at 4°C, followed by a wash with 3 mL of FACS buffer (spinning at 500*g* for 5 minutes). The secondary antibody goat anti-human IgE–fluorescein isothiocyanate (FI-3040; Vector Laboratories, Burlingame, Calif) was incubated at 30 μg/mL in FACS buffer for 30 minutes at 4°C, followed by one wash as above. Samples were resuspended in 100 μL of FACS buffer and analyzed with a FACSCanto II (BD Biosciences, San Jose, Calif).

### Purification of CSPG4 IgE

CPSG4 IgE was purified with HiTrap KappaSelect columns (GE Healthcare, Fairfield, Conn), according to the manufacturer's instructions. Briefly, columns were equilibrated with 10 column volumes of PBS, cell-culture supernatant was diluted 1:1 vol/vol with PBS, and samples were loaded on the column followed by a wash with at least 20 column volumes of PBS. The sample was then eluted with 5 column volumes of 0.1 mol/L glycine buffer at pH 2.3 and immediately buffered to pH 7.5 by using 1 mol/L Tris, pH 9.0. Purified antibodies were then dialyzed against PBS overnight at 4°C and sterilized with a 0.2-μm sterile filter.

### Size exclusion chromatography

Purified antibodies were analyzed by using size exclusion chromatography, as previously described.^E3^ Briefly, gel filtration was performed on a Gilson HPLC system using a Superdex 200 10/300 GL column (GE Healthcare), which is suitable for purifying proteins between 10 and 300 kDa at a flow rate of 0.75 mL/min in PBS (pH 7.0, 0.2 μm filtered).

### Lectin blot

Purified IgE samples (150 ng) were reduced with 50 mmol/L dithiothreitol and boiled at 95°C for 5 minutes. Samples were run at 150 V on Mini-PROTEAN TGX Gels 4-15% (Bio-Rad Laboratories, Hercules, Calif) and blotted with Trans-Blot Turbo Transfer Pack PVDF (Bio-Rad Laboratories) by using the Trans-Blot Turbo Blotting System (Bio-Rad Laboratories), according to the manufacturer's instructions. The blotted membrane was then cut just above 35 kDa to have heavy (50 kDa) and light (25 kDa) chains in different membranes. The heavy chain membrane was blocked with Carbo-Free Blocking Solution (Vector Laboratories) for 1 hour and then probed with RCAI-biotin (*Ricinus communis* agglutinin I lectin [Vector Laboratories] specific for galactose), AAL-biotin (*Aleuria aurantia* lectin [Vector Laboratories] specific for fucose), or Con-A–biotin (concanavalin A lectin [Vector Laboratories] specific for mannose) at 0.2 μg/mL in Carbo-Free Blocking Solution for 30 minutes. The membrane was then washed 3 times in PBS–Tween 0.05% (T-PBS) and incubated with High Sensitivity Streptavidin-HRP (1:30,000; Pierce) for 30 minutes, washed as above, and developed with ECL (Amersham, GE Healthcare). The light chain membrane was blocked with T-PBS and 5% BSA for 1 hour at room temperature and then incubated overnight at 4°C with rabbit anti-human kappa light chain antibody (1:1,000 in T-PBS 5% BSA; Abcam, Cambridge, United Kingdom), followed by 3 washes in T-PBS. The membrane was incubated with anti-rabbit IgG horseradish peroxidase antibody (1:2,000 in T-PBS and 5% BSA; Cell Signaling Technology, Danvers, Mass) for 1 hour at room temperature, washed as above, and developed with ECL. Densitometric quantification was performed with ImageJ software (National Institutes of Health, Bethesda, Md), and values were normalized by the loading control (kappa light chain) and for the value obtained in 0.5M/mL 4D culture conditions.

### PNGase F release of N-glycans

IgE samples were dried down before release. PNGase F release of N-glycans (E-PNG01; QA-Bio, http://glycotools.qa-bio.com) was performed in a manner similar to that previously published.^E4^ In short, 25-μg aliquots of the samples in water (17.5 μL) were incubated with 5 μL of 5× Reaction Buffer 7.5 and 1.25 μL of Denaturation Solution for 10 minutes at 100°C, followed by addition of Triton X-100 (1.25 μL) and PNGase F solution (1 μL) and incubation overnight at 37°C. Dried-down samples were treated with 20 μL of 1% formic acid for 50 minutes at room temperature, followed by removal of protein material with a Protein Binding Membrane plate (LC-PBM-96; Ludger, Oxfordshire, United Kingdom).

### Procainamide labeling and cleanup

Released glycans were labeled with procainamide by using a procainamide labeling kit (LT-KPROC-24; Ludger) in a manner similar to that previously reported.^E5^ In short, 150 μL of 30% glacial acetic acid in dimethyl sulfoxide was added to a vial of procainamide, followed by 150 μL of water. This solution was transferred to a vial of sodium cyanoborohydride to make the final labeling reagent. Twenty microliters of labeling reagent was added to each sample. The samples were then incubated for 1 hour at 65°C. An HILIC method was performed to clean up the samples and remove free dye by using an LC-PROC-96 plate (Ludger) on a vacuum manifold. Samples were eluted from the cleanup plate in 300 μL of water.

### LC-MS analysis of procainamide-labeled N-glycans

Samples were analyzed by using HILIC-LC on an Ultimate 3000 UHPLC with a BEH-Glycan 1.7 μm, 2.1 × 150 mm column (Waters, Milford, Mass) at 40°C with a fluorescence detector (λex = 310 nm, λem = 370 nm) controlled by Bruker HyStar 3.2 (Bruker, Billerica, Mass). Buffer A was 50 mmol/L ammonium formate made from LudgerSep N Buffer stock solution, pH 4.4 (LS-N-BUFFX40); buffer B was acetonitrile (acetonitrile 190 far UV/gradient quality; Romil #H049). Samples were injected in 25% aqueous/75% acetonitrile; injection volume was 25 μL. Separation was performed with a linear gradient of 76-53% ACN at 0.4 mL/min in a 71-minute analytic run. PROC-labeled glucose homopolymer was used as a system suitability standard, as well as an external calibration standard, for glucose unit allocation for the system. Chromeleon Data Software (version 7.2; Thermo Fisher Scientific, Waltham, Mass) with a cubic spline fit was used to allocate glucose unit values to peaks. A Bruker amaZon Speed ETD electrospray mass spectrometer (Bruker) was coupled directly after the ultra-HPLC with fluorescence detection without splitting. The instrument scanned samples in maximum resolution mode with a positive ion setting, mass spectrometry (MS) plus 3 MS/MS scans, nebulizer pressure of 14.5 psi, nitrogen flow of 10 L/min, and capillary voltage of 4500 V. MS/MS was performed on 3 ions in each scan sweep with a mixing time of 40 ms. MS data were analyzed by using Bruker Compass DataAnalysis 4.1 software.

### Binding of CSPG4 IgE to tumor and immune cell lines

Binding of CSPG4 IgE to A375 and RBL-SX38 cells was performed by using 10^5^ cells per sample. Cells were detached by using PBS plus 5 mmol/L EDTA. For each primary antibody, serial dilutions were prepared in FACS buffer (PBS and 2% FBS). Primary antibodies were incubated for 30 minutes at 4°C, followed by a wash with 3 mL of FACS buffer (spinning at 500*g* for 5 minutes). Samples were incubated with the secondary antibody goat anti-human IgE–fluorescein isothiocyanate (FI-3040; Vector Laboratories) at 30 μg/mL in FACS buffer per sample for 30 minutes at 4°C, followed by 1 wash as above. Samples were resuspended in 100 μL of FACS buffer and analyzed with a FACSCanto II.

### RBL-SX38 degranulation assays

We evaluated degranulation of RBL-SX38 cells by quantifying β-hexosaminidase release, as previously described.E6, E7 Control conditions used were as follows: unstimulated cells; Triton X-100 lysed cells (to quantify 100% granule release); chimeric NIP-IgE against the hapten 5-iodo-4-hydroxy-3-nitrophenyl; unstimulated or NIP-IgE–stimulated cells plus polyclonal rabbit anti-IgE (Dako), or antigen-expressing tumor cells as cross-linkers. Cells were seeded at 1 × 10^4^ cells per well in culture medium overnight. The next day, cells were sensitized with IgE (200 ng/mL), control antibody, or medium alone by incubating them at 37°C for 2 hours. Cells were then washed 3 times in stimulation buffer (HBSS and 1% FBS; Gibco) and stimulated for 45 minutes at 37°C with 100 μL of stimulation buffer, rabbit anti-IgE (1.5 μg/mL), or 3 × 10^4^ CSPG4^high^ A375 tumor cell line. To detect β-hexosaminidase, 50-μL culture supernatants diluted 1:1 in stimulation buffer were transferred onto black 96-well plates with 50 μL of florigenic substrate per well (1 mmol/L 4-methylumbelliferyl N-acetyl-b-D-glucosaminide, 0.1% dimethyl sulfoxide, 0.1% Triton X-100, and 200 mmol/L citrate, pH 4.5) and incubated for 2 hours in the dark at 37°C. Reactions were quenched with 100 μL per well of 0.5 mol/L Tris, and plates were read with a FLUOstar Omega Microplate Reader (350-nm excitation and 450-nm emission; BMG Labtech). Degranulation was expressed as a percentage of Triton X-100 release (100%) and compared with that in unstimulated cells (0%).

### Evaluation of antibody-specific productivity

Expi-CSPG4-IgE cells were cultured in different conditions, and IgE secretion and cell viability were monitored daily. Antibody-specific productivity (*q*_*mAb*_ [pg cell^−1^ d^−1^]) was calculated according to the following equation^E8^:

$$q_{mAb}=\frac{m_{mAb}}{\frac{\left(N-N_{0}\right)\;\times \;t}{\log_{e}\left(N/N_{0}\right)}}$$

with *m*_*mAb*_ as secreted IgE; *N* and *N*_*0*_ as the final and initial viable cell values, respectively; and *t* as days in culture.

### Statistical analysis

Error bars represent SDs or SEMs. Statistical significance of the degranulation assay was calculated with 1-way ANOVA with the Dunnett multiple comparisons test. *P* values of less than .05 were considered statistically significant as follows. Data were analyzed with Prism 7 software (GraphPad Software, La Jolla, Calif).
